# The lived experiences of pregnant women during COVID-19 pandemic: a descriptive phenomenological study

**DOI:** 10.1186/s12884-021-03691-y

**Published:** 2021-03-08

**Authors:** Forough Mortazavi, Fatemeh Ghardashi

**Affiliations:** 1 Noncommunicable Diseases Research Center, Sabzevar University of Medical Sciences, Pardis Building, Towhid Blvd, Sabzevar, Iran; 2 Noncommunicable Diseases Research Center, Sabzevar University of Medical Sciences, Sabzevar, Iran

**Keywords:** Qualitative research, Phenomenology, Women, Pregnancy, COVID-19

## Abstract

**Background:**

With the onset of the COVID-19 epidemic, pregnancy and childbirth for women are taking place in unusual circumstances. We explored the lived experiences of pregnant women during the COVID-19 pandemic to better understand their experience of pregnancy so that better support could be provided.

**Methods:**

We used a descriptive phenomenological approach to understand the lived experience of pregnant women in COVID-19 pandemic. We collected data using a purposive sampling method through in-depth interviews in cyberspace with a semi-structured questionnaire. We used Colaizzi’s seven-step content analysis method to analyze the research data with the help of MAXQDA software version 2020.

**Results:**

We conducted this descriptive phenomenology study on 19 pregnant women in a period between the 10th to the 20th of May, 2020. The participating women were already pregnant when the first signs of the epidemic appeared in the country and at the time of the interview. We acquired four themes including disruption of the tranquility and regular routines of daily life, new challenges caused by the epidemic, resilience and strength in facing the crisis, and adaptation with new conditions.

**Conclusions:**

The pregnant women were under intense stress during the COVID-19 outbreak. The general mobilization the health system is necessary for alleviating pregnant women’s difficulties in situations like the COVID-19 epidemic. Virtual training classes and virtual counseling may enhance the peace and tranquility of pregnant women.

**Supplementary Information:**

The online version contains supplementary material available at 10.1186/s12884-021-03691-y.

## Background

Pregnancy is one of the most pleasant and at the same time most critical periods in the life of most women. It involves a host of new and unprecedented emotions and experiences. Unfortunately, with the onset of the COVID-19 epidemic, pregnancy and childbirth for women are taking place in utterly new and unusual circumstances.

A number of issues have led to a state of confusion and anxiety among pregnant women in the current crisis. Among these the most concerning are the continuous stream of news reports about rising number of infections and the increasing number of severe cases and death [1]. Other concerning issues are the emotional, physical, and mental exhaustion and loss of medical staff, the shortage of essential hygiene equipment, the varieties of symptoms and secondary diseases caused by the infection, and the failure of many proposed treatments [2]. Furthermore, the dissemination of false information on online platforms has played a part in creating high levels of fear and anxiety among pregnant women [3]. Apart from concerns affecting the general population, there are specific issues causing higher levels of anxiety among pregnant women. These include issues such as higher risk of contracting COVID − 19, vulnerability to severe complications [4], the risk of death [5], the risk of mother-to-child transmission, and the potential effects of COVID-19 on the fetus [6].

Studies show that in previous epidemics, pregnant women have been among the most vulnerable groups of people and have suffered badly [7, 8]. During the severe acute respiratory syndrome (SARS) outbreak in Hong Kong, pregnant women reported frustration, anxiety, problems with sleeping, and disruption of their daily lives [9]. Women were reluctant to attend hospitals for fear of infection. They also experienced insecurity and uneasiness even at home and had worries about contracting the disease [10]. Hospitals in Taiwan did not provide prenatal care and women were discharged early after childbirth. There was also a significant increase in the number of cesarean [11]. In the H1N1 pandemic, pregnant women reported worries about the future, their health and the risk of their babies and close acquaintances being infected by the virus. Some women felt it was not safe to go to work anymore and stayed at home [12]. In the Zika virus epidemic, women were affected by feelings of anxiety, helplessness and mistrust, and uncertainty about the new disease [13]. In the Ebola epidemic, women avoided going to health centers because they were afraid of contracting the disease. Health facilities also refused to provide services to pregnant women because of the risk that they might have carried the virus [14]. So, similar problems were expected to occur for pregnant women as a result of the COVID-19 epidemic. Fear, anxiety, and worry during pregnancy has negative health consequences for both pregnant women and the growing fetus [15, 16].

Qualitative research is a systematic approach to describing individuals’ experiences and what lies behind those experiences to give them meaning [17, 18]. Phenomenology is a way to discover the individuals’ lived experiences or the world of life [19]. The “world of life” is an experience that is achieved without voluntary thinking and usually includes things that are taken for granted or things that are common [20].

Since the outbreak of COVID-19, discussions in the field of obstetrics have mostly focused on the pregnancy complications in infected women and the probability of mother-to-fetus transmission. So far, the lived experience of infected pregnant women with COVID-19 has been investigated [21]. The experience of women who were pregnant but not infected has not been investigated. Therefore, the purpose of this study is to understand lived experiences of pregnant women during the COVID-19 epidemic in such areas as their mental state, the effects of the epidemic on their personal life, the experience of home quarantine and the impact of the COVID-19 pandemic news on their health. Investigating these matters will help to better understand their experience of pregnancy during the COVID-19 epidemic so that better support could be provided in case the present epidemic continues or similar ones occur. We conducted this study on pregnant women in Iran because it has been severely affected by COVID-19 pandemic [22] which broke out in the country in Feb, 2020.

## Methods

We used a descriptive phenomenological approach to understand the lived experience of pregnant women in COVID-19 pandemic. In this approach, the researcher investigate the meaning and concept of a phenomenon from the participants’ perspective.

### Participants and setting

We conducted this qualitative study on pregnant women who were registered in public health centers affiliated with Sabzevar University of Medical Sciences operating in urban sites in Sabzevar, Northeast of Iran. Data was collected using a purposive sampling method through in-depth interviews in cyberspace with a semi-structured questionnaire. We interviewed women in the period between the 10th to the 20th of May, 2020. The rationale for the sample size in this qualitative study was obtaining a diverse sample and having sufficient textual data to make an iterative categorization of qualitative data possible. Four midwives each working in one of four health centers in different socio economic areas of the city contacted eligible pregnant women registered with them to invite them to participate in the study. We instructed midwives to choose a varied sample of participants including both housewives and employed women, primiparous and multiparous women, and women with different education levels.

The length of each interview was 25–30 min in the WhatsApp social network. A midwife attended each session as a facilitator. Overall, 19 women were interviewed. We conducted a virtual interview to observe hygiene protocols. Inclusion criteria were being pregnant at the time of announcement of epidemic in Iran and at the time of interview, and being consent to participate in the study. Individual interviews started with questions about women’s experience in the first days after the official announcement of COVID-19 outbreak in Iran. It was continued with questions about their experience of home quarantine, following of COVID-19 news, and the effects of the news on their lives. We developed the interview guide for this study based on our experience with pregnant women during the early stages of COVID-19 outbreak in Feb 2020 (supplementary file). We used data analysis results of each interview as a guide for the next interview. Sampling was performed continually until data saturation and to the point that no new code could be extracted.

### Data analysis

We used Colaizzi’s seven-step content analysis method [23] to analyze the research data with the help of MAXQDA software version 2020 (VERBI Software GmbH, Berlin, Germany). At the end of each interview, conversations were copy-pasted from WhatsApp social media to a word file. In the first stage, we read the files several times to understand women’s feelings and experiences. We tried to suspend our previous thoughts, feelings, or ideas (bracketing) about the phenomenon under study. In the second stage, we identified important phrases in the text of the interviews. Then in the third step, we extracted the concepts and in the fourth, we categorized concepts into classes based on the similarity of the concepts. In the fifth stage, we combined the results to describe the phenomenon under study in terms of categories that are more general. In the sixth stage, we presented a comprehensive description of the structure of the phenomenon under study. In the final stage, we validated the structure by comparing it to the experiences of the participants.

### Credibility& reliability

In addition to bracketing, two researchers read the files several times and performed data analysis independently. Then, we discussed codes, themes clusters, and themes in case of discrepancy. To increase credibility, we allocated a long time to engage in the details of the interviews and analysis of contradictory cases. We examined the similarity of the extracted themes and themes clusters to those extracted by an observer. For this purpose, we printed the interviews and presented them to an outside observer who is an expert in qualitative research. There was 85% agreement in coding and theme extraction between the researchers and the observer. In case of disagreement, we reviewed the data and analyzing the disagreement.

### Ethical considerations

Women who agreed to participate in the study received an informed consent form via text message. We interviewed the women who had read the informed consent form and had communicated their consent to participate in the study by responding with an “I consent” text message. We informed women that they could participate in the study by changing the name of their profile to a pseudonym and responding to the questions by typing their answers. After each interview, we removed the participant from the group, and cut-pasted the interview conversations in a Word file.

## Results

This descriptive phenomenology study was conducted on 19 pregnant women in a time-frame between the 10th to the 20th of May, 2020. The mandatory quarantine period (20th of March to the 3rd of April) had come to an end by the time the study was about to begin. The participating women were already pregnant when the first signs of the epidemic (19th of February) appeared in the country. Table 1 shows the specifics of the participating samples. In one of the interviews, one of the pregnant women stated: “I can seriously say that my anxiety and fear has doubled and includes anything and everything that could come to a person’s mind.” We directed her to a psychiatrist due to the possibility of mental illness. We acquired four themes including disruption of the tranquility and regular routines of daily life, new challenges caused by the epidemic, resilience and strength in facing the crisis, and adaptation with new conditions (Table 2).

**Table 1:** Participants’ characteristics

Variable	M ± SD	Minimum	Maximum
Age (year)	29.3 ± 4.0	24	37
Education (year)	15.5 (1.7)	12	18
Gestational age	28.4 (6.4)	16	38
Parity	N(%)		
Nullipara	12 (63.2)		
Para 1	5 (26.3)		
Para 2	2 (10.5)		
Job			
Employed	5 (26.3)		
Housewife	14 (73.7)

**Table 2:** Theme categories and clusters

Themes	Clusters
1. Disruption of the tranquility and regular routines of daily life	
Emotional responses	
	A. Intense stress
	B. Fear of infection
	C. Concerns
	D. Sense of loneliness and lack of support
	E. Depression and loneliness in quarantine
Behavioral responses	
	A. Practical obsessions
	B. Change in nutrition
	C. Adhering to quarantine
	D. Adhering to sanitarian protocols
	E. Following the news
2. New challenges caused by the epidemic	
	A. Problems in acquiring health products
	B. Disruptions in receiving health-care
	C. Cancellation childbirth preparatory classes
3. Resilience and strength in facing the crisis	
	A. Creation of a WhatsApp social media group
	B. Holding virtual childbirth-preparatory classes
4. Adaptation with new conditions	
	A. Reduction in stress
	B. Regulations in the levels of adherence the sanitarian protocols

### Theme 1. Disruption of the tranquility and regular routines of daily life

This theme consists of five sub-themes in the areas of psychological responses related to stress, fear, anxiety, depression, loneliness, and lack of support from related organizations. We found five Sub-themes in the area of behavioral responses, including practical obsession, adhering to the quarantine and adhering to the sanitarian protocols, changing lifestyle, and following the news.

#### Intense stress

The pregnant women were under intense stress for the first few weeks after the official confirmation of the epidemic in the country. This stress rose as the infection and mortality rates increased.

“In the beginning I wouldn’t even let my husband go to work. I was afraid of shopping or anything else that required contact with other people.”

“I was highly stressed and did not leave the house for a month. The only place I visited was my father’s house in Mashhad, because I was assured that they too were in self-quarantine.”

“I was not so stressed in the beginning but became so as the mortality rates increased.”

#### Fear of infection

The pregnant women were highly afraid of becoming infected in the time of their pregnancy and childbirth. Since the health of a fetus and its mother is inseparable, this fear was mostly for the safety and health of the former. The women were afraid of busy places such as hospitals, of miscarriage due to the corona-virus, and of their children or companions becoming infected in the hospitals.

“It’s because no matter how careful you are, you’ll still be in close contact with the nurses and the midwives … especially the infant.”

“I was in my 5^th^ month in March when I saw a clip of infected womens who had successfully given birth and were being treated. I was constantly stressed by not know what would happen to my unborn baby if I were to get sick. Would I have to abort? Would my child become sick too?”

“I would even cry whenever I’d hear that a newborn was infected. I didn’t want this to happen to me.”

“I am in the 38th week and I am worried about the hospital. I heard that the virus has involved all the hospitals. I am worried that my baby, my companion, or I would get the infection.”

#### Anxiety

The pregnant women were aware of the effects of stress in times of pregnancy. They had heard that a pregnant woman is more vulnerable towards COVID-19 compared to others. In some of the cases, pregnancy itself was high risk for the women, and this would worry them. Another source of distress for women was that, since the start of the pandemic, hospitals no longer allowed entry to companions and family members. The pregnant women also experienced other worries (as experienced by non-pregnant individuals), such as their husbands or older and sickly family members becoming infected. In some ways, the worry for the husbands becoming infected was because of the close nature of husband/wife relationship, and in reality a worry for the woman herself to get infected as a result. Some were also thinking about the possibility of the infection to pass to the infant through breastfeeding.

“What can we do with so stress these days? With these stresses, how we can be healthy women and give birth to a healthy child.”

“Is it true that pregnant women are more vulnerable to this infection? Are they immune system deficient?”

“I take care of myself but my husband go out. What can I do if he gets the virus and transmits it to me?”

“Right now my anxiety about corona is due to me having to be alone during childbirth. I wish that it would not continue.”

“This is my first pregnancy after trying for ten years. Maybe this why I am so worried.”

“I’m more concerned for my husband and my parents than myself. I was in home-quarantine but my husband had to leave every day for work. His job has him be in contact with many people.

“My husband would wear a mask whenever he left the house, but he didn’t care as much as I did, and that would add to my worries.”

#### Sense of loneliness and lack of support

The pregnant women felt that they were not being sufficiently supported during this epidemic. They complained of the closing of the clinics, the push to make women avoid visiting health centers, and the lack of health-package allocation to pregnant women.

“The thing that I was unhappy with the most was that no organization provided support to pregnant women. I expected the Health Ministry to be more caring to high-risk individuals such as pregnant women, but they didn’t even give us masks.”

“I couldn’t find any masks when I had to go to the hospital around the 8^th^ of April. They didn’t care enough to provide us with any, but would remind us that we must not enter without a mask.”

“Most of the physicians closed down their clinics. They are still closed. I was forced to visit the doctors in the hospitals. I was in that unclean environment from 9:00 to 13:00. The hospital is supposed to care for pregnant women, but unfortunately, it is not. I expected the hospital beds to have disposable sheets, used only once for each individual. There’s not even enough room there to sit down. They wouldn’t let us in the hallways and said that we had to wait in the yard. The seats were all filled by people who had accompanied other patients, sometimes from out-of-town.”

#### Depression and loneliness in quarantine

Although quarantine had resulted in the decrease of stress and obsession in the women, it had also resulted in the experience of loneliness and depression.

“Being imprisoned in the house and not being able to visit my family and friends for a month made me feel depressed and agitated.”

“I didn’t leave the house at all. I was going crazy with depression. I was sick of the house because I was alone.”

“My lifestyle was moving towards increased loneliness. I mean, I was becoming a solitary person because I had not seen any other people for so long. My family do not live in this city. I was very lonely.”

#### Practical obsessions

The women themselves felt that they had become more obsessive in thought. This obsession manifested itself practically in repeatedly disinfecting surfaces, washing hands, fruits and vegetables, and a heightened sensitivity towards their husbands adhering to the sanitation protocols.

“I overdid it in the beginning. It was as if something had possessed me, and forced me to disinfect the house several times every day. My hands were completely ruined.”

“I was constantly reminding my husband. Constantly asking him about whether or not he has washed his hands is annoying to the both of us.”

“Certainly, everything becomes an obsession. Even now, I wash every fruit I buy and package it in clean plastic bags, and then wash them again before eating.”

#### Change in nutrition

The nutritional intake of the pregnant women changed based on suggestions that were common in the beginning of the epidemic. Some of these changes included: abstaining from consuming bulk foodstuff and food from the outside (restaurants etc.), and consuming more cooked food and specific fruits and vegetables.

“Lemon consumption increased. We used to buy yogurt from local shops, but now we only buy the large packages from companies. We eat more cooked food. We no longer buy pickles and cheese in bulk. I make my own pickles, and we only buy pasteurized cheese.

“There was a lot that I wanted to eat outside but I couldn’t because of the virus. I still haven’t at all.”

#### Adhering to quarantine

The pregnant women adhered to the quarantine very well. They stated that doing so had reduced their stress and obsessive behavior. Although it is common for Iranians to travel and visit their families during Now’Ruz (New Years), the pregnant women stayed at home and did not go on holidays.

“There was no New Year’s meet-and-greet this time around. I haven’t let anybody come to our house for the past 3 months. We only keep in-contact via telephone.”

“Me, my husband, my parents and my brother were all in quarantine outside the city and away from everybody else. I did not have any Corona-related stress because I closely followed the quarantine rules. For 2-3 months, I wouldn’t even roll down my car windows whenever I was in the city.”

#### Adhering to sanitarian protocols

Sanitarian protocols quickly went viral on the television and social media and the women would follow and adhere to them in detail.

“My husband and I closely follow the rules and always wear masks and gloves, and carry disinfectants with us.”

“In the beginning I would bring my own sheets to cover the beds in the sonography and other clinics. Sometimes I would even use my chador (veil covering the entire body) and then wash it once home.

“I’ve put a container in a corner of the kitchen and I put everything that comes from the outside there first so they are not in contact with anything else. Then I start washing them.”

#### Following the news

Most of the pregnant women initially followed the news via virtual (social networks) and official (television) channels but later avoided doing so – especially in the case of social media networks to reduce stress and anxiety. Most of the women expressed lack of faith in the truthfulness of statistics provided by the Ministry of Health.

“I was always waiting for news and constantly followed it whether on the TV or social media because they were important to me.”

“I decided to pay less attention to the news after a while because it caused me a lot of anxiety.”

“My stress increased gradually and I was constantly worried of losing someone which really depressed my mood. It got much better once I abstained from social media for a while, but usually a single piece of bad news was enough for me to feel down for the rest of the day. I mostly followed the news via television, even though I knew the statistics provided is false. But at least they didn’t exaggerate things morbidly like they do on social media.

“I saw on the internet and satellite TV that the pandemic has reached Iran. Watching videos of people falling on the ground caused me great fear. I experienced a lot of stress and decided to abstain from social media for a while. It made me feel better.”

### Theme 2. New challenges caused by the epidemic

The epidemic caused a lot of problems for the pregnant women such as the push to prevent people from visiting health centers, the closing-down of some clinics, the week-long New Year’s holidays, and the shortage of health products.

#### Problems in acquiring health products and lack of facilities

In the beginnings of the epidemic, there was a noticeable shortage of health products such as hygiene gels and masks in the hospitals, health centers, and pharmacies.

“Initially I could not find any masks at all. I think the Health Ministry should have provided a package including these items just like it does with the drugs it provides pregnant women.”

#### Disruptions in receiving health-care

Women mentioned some disruptions in receiving health-care in the beginnings of the epidemic such as cancellation of pregnancy-related appointments, the closing-down of some specialist private offices, the wait-times for visits in hospitals, and the crowds of visitors in sonography and other types of clinics.

“The fear, stress and the warnings by the health ministry have made me no longer visit my doctor or other health centers. I’ve been having cramps for two weeks and it’s getting worse. I’m afraid of giving birth pre-maturely.

“The clinic overseeing me is very busy and they don’t follow the protocols. They won’t give appointments by the telephone either. It always bothers me and my back gets hurt”.

#### Cancellation childbirth preparatory classes

Childbirth preparatory classes have become common in recent years. Usually, after attending the classes, the women could select their own midwives. The cancellations of the childbirth preparatory classes initially worried the pregnant women who looked forward to them.

“All the preparatory classes by the hospitals got cancelled and there were talk of us having to wait until after the New-Year’s holidays for things to go back to normal. We waited but things did not change.”

“With the closure of the classes, we do not learn anything; so, at the time of labor in the hospital, midwives would say that you do not know anything.”

### Theme 3. Resilience and strength in facing the crisis

#### Creation of a WhatsApp social media group

Pregnant women’s responses to the creation of the whatsApp online group were generally positive and welcoming. This group comprised of pregnant women, midwives from health centers, and some faculty members.

“There was a counseling group and you could ask questions, which is ideal and great. In my opinion, it should continue even after Corona. I read the content and trust it since it’s written by experienced midwives. I feel comfortable knowing that the information is from a verified source. Also, you can always ask questions from the midwives or other women like yourself.”

“I’ve only been a member for a 2-3 days but it has still been useful to me in this short time. I’m very grateful that you answer our questions and it’s great to know that the person answering them is an experienced.”

#### Holding virtual childbirth-preparatory classes

Women found the idea of online preparatory classes useful and were keen to participate in them.

“The most important event that occurred in the time of the Corona were the virtual classes. They greatly increased my understanding and gave me hope, since I was already looking for information. I did the exercises slowly and carefully. You could even save the videos so you don’t forget the small details.

“I definitely think it was necessary and I really appreciated it. Thank God the Corona couldn’t take this away from us.”

“Virtual classes are easier and they won’t waste your time, but of course the quality is better in-person. Still, it’s better for the pregnant women who cannot sit for long.”

### Adaptation with new conditions

#### Reduction in stress

The findings show that the level of stress has decreased as time passed. Some of the women spoke of reductions in their stress levels and obsessive behavior.

“I don’t worry much about getting infected because I follow the guidelines.”

“My obsessions have subsided significantly, because I spend most of the time at home. First off, I follow the sanitation guidelines, and next I’ll continue living a stress-free life with the help of God.”

#### Regulations in the levels of adherence the sanitarian protocols

Due to the reduced stress, most of the women have revised their obsessive adherence to the sanitarian protocols.

“I relaxed a bit after Eid-e-Fitr. I had previously quarantined myself, but now I leave the house, albeit by following the protocols. I even allow some guests to visit us, but I always give them disinfectants to clean their hands first-thing.

“I no longer have the same fear, though the cleanliness habits have remained.”

## Discussion

This qualitative study explored pregnant women’s experiences during the COVID-19 epidemic. We extracted 4 themes and 17 sub-themes from conversations with the women. The results showed that pregnant women experienced hardships and severe stress in their daily lives during the first weeks of the official announcement of the epidemic and the quarantine period in Iran. Stress, fear, worry and anxiety, feelings of depression, and loneliness were common among pregnant women during the epidemic. According to other studies, most of these responses were also present in other groups during the COVID-19 epidemic crisis [24–27]. Similar responses were also reported in pregnant women during the previous epidemics and pandemics [9, 10, 12, 14].

Pregnant women when compared to other groups, have other concern in addition to their own health. They are concerned about the health of the fetus and having a healthy delivery. Studies indicated that a higher percentage of pregnant women experienced extreme fear, anxiety, and depression during the COVID-19 epidemic compared to normal times [28–31]. The main concern of women in this study was the possibility of being infected with COVID-19 and transferring the virus to the growing fetus. Results of two studies in Iran indicated that the possibility of giving birth to an abnormal/unhealthy baby was the most prevalent causes of worry and anxiety in pregnant women in normal conditions [32, 33]. Similar concerns were reported during the previous epidemics and pandemics [9, 10, 12, 14].

We found that women sought to reduce their risk of infection through behavioral responses such as strict adherence to quarantine and health protocols and lifestyle changes. Behavioral changes usually happen as a result of psychological experiences such as stress, fear and worry [34]. According to the previous studies, pregnant women used behavioral strategies to lower their risk of getting SARS and Zika virus [9, 10, 13].

At the beginning of the epidemic, the women followed the news about the epidemic on social media and on the Iranian state television, but they stopped following the news and in particular, they stopped following the news on social media altogether when disappointing news led to more stress and anxiety. The available literature shows that when a message is disseminated through the media, the most important issue is how it is presented. It may heighten or lower the audience’s perception of the severity of the situation depending on the framework adopted for coverage [35]. The news media and in particular the social media played a negative and harmful role at the beginning of the COVID-19 pandemic [36]. Their coverage spread more distress and anxiety rather than more awareness and readiness for confronting the situation. There was also a large measure of distrust among most of the women with regard to the official statistics of COVID-19 cases announced by the Health Ministry. The result of Samadipour and Ghardashi’s (2020) online study indicate that more than 80% of participants in 20 provinces of Iran were distrustful of the authorities [25].

It is a cultural norm of Iranian society to provide high level support and care to pregnant women. Proverbs such as “a pregnant woman carries a load of glass” indicate that the community cares for the health of pregnant women. The findings of the present study show that, during the COVID-19 epidemic, pregnant women felt unsupported, lonely, and abandoned due to the lack of community and family support. Women could not be accompanied to hospital for childbirth. As a result they felt unsupported and helpless and under more stress. The COVID-19 pandemic caused a number of changes in the way prenatal care is provide. These changes as reported in a previous study included cancellation of appointments and also restriction on support persons accompanying women during visits to health centers [28]. The Yue’s study in China also found that community support had a direct effect on reducing maternal anxiety levels [37]. It is unfortunate that in times of crisis and trouble certain groups of people are left unattended and have to endure intolerable pain and suffering on their own because public health authorities have to focus on a swift response to the crisis.

To cope with the various challenges pregnant women were facing, some faculty members and midwives formed a group on the WhatsApp social network to provide pregnant women with advice and up-to-date information on COVID-19. We also held virtual childbirth preparatory classes in response to requests by the women. Crisis management has four stages: prevention, preparedness, response and recovery. Due to unpredictable and rapid spread of COVID − 19 disease, there can really be no meaningful prevention phase. Also in many countries, the authorities were unprepared for a crisis of this magnitude or if they had a “pandemic playbook” they failed to follow it. Therefore, what they were able to do was to adopt basic measures to respond to the crisis and to ameliorate the situation and start the work of restoring the normal conditions.

Over time, women’s stress levels and obsessive behaviors decreased. As reason for this, we can point to reductions in the rate of infections and the death toll after the mandatory quarantine period and the re-opening of the clinics and the resumption of visits to health centers. In addition, the re-opening of businesses and the relative normalization of life might have been effective in decreasing the levels of stress. It is also likely that the support provided to women during the first wave of the disease was effective in reducing their stress level. Lee and colleagues investigated the psychological responses of pregnant women to the SARS outbreak in Hong Kong. They found that pregnant women’s depression rate during the SARS epidemic was not different from the pre epidemic rate. This was probably due to the support provided to the women during the SARS epidemic [10].

Coping refers to cognitive and behavioral efforts to cope with difficult situations. In confronting stressful situations, individuals usually adopt two main types of coping strategies: problem-focused and emotion-focused. Proper adaptation and coping strategies are usually problem-oriented. They usually involve looking for alternative solutions to newly encountered problems, and also looking for new ways of obtaining community support to protect oneself and others [38]. It seems that, women in our study adopted problem-focused techniques to cope with COVID-19 pandemic.

Participants in this study consisted of relatively affluent and educated women. Women from poorer backgrounds or with low education levels and those who did not have a smartphone of their own or those whose phone was turned off and failed to reply to repeated attempts for contact could not be invited to participate in this study. In addition, several women did not respond to the invitations or did not agree to be interviewed. They might have been unwilling to participate because they were experiencing higher levels of stress, anxiety, and depression. Those women may have different stories of loss of a family member or facing the economic consequences of the epidemic. Another limitation of our study was that we had to conduct the interviews online because of the risk of infection; so, we could not observe non-verbal responses from women. Therefore, these limitations must be considered in generalizing the findings on this population to all pregnant women.

Online interviews have theirs strengths and weaknesses. Shy people are more forthcoming and willing to share their experiences in such interviews. They also save time and travel costs for both interviewers and interviewees. One of the weaknesses of this study is that we interviewed women after the first wave of the epidemic and asked them to recall their experiences during the previous month, and this may be subject to recall bias. It is also likely that they were not able describe the psychological impact of COVID-19 outbreak on themselves precisely.

## Conclusions

In this phenomenological study, we explored pregnant women’s lived experiences during the COVID-19 pandemic. We extracted 4 themes and 17 subthemes from our interviews. The main themes were disruption of the tranquility and regular routines of daily life, new challenges and problems caused by the epidemic, resilience and strength in facing the crisis, and adaptation and coping with new conditions. The lack of community support in situations like the COVID-19 epidemic necessitates the general mobilization the health systems for alleviating pregnant women’s difficulties. Further research is needed to examine different supportive measures for enhancing peace and tranquility of pregnant women in difficult conditions. Considering the socio economic background of the participants, the findings of this study can only be generalized to the relatively affluent and educated pregnant women population.

## Supplementary Information

**Additional file 1.**

## Data Availability

The data that support the findings of this study are available from the corresponding author upon reasonable request and with permission of Sabzevar University of Medical Sciences.

## Abbreviation

COVID-19: Corona Virus Disease-2019
