# Supplementary material for: The lived experiences of pregnant women during COVID-19 pandemic: a descriptive phenomenological study
Source: BMC Pregnancy Childbirth. 2021 Mar 8;21:193. doi: 10.1186/s12884-021-03691-y (PMC7938285; doi:10.1186/s12884-021-03691-y)
Supplement: Supplementary file 1 — Additional file 1. [file 12884_2021_3691_MOESM1_ESM.docx]

**Interview guide**

1. What was your experience in the first days after the official announcement of COVID-19 outbreak in Iran?

1.1. Please describe your concerns.

1.2. Please talk about your fears.

1.3. Please talk about your problems.

2. What was your experience of home quarantine?

2.1. Please describe your concerns.

2.2. Please talk about your fears.

2.3. Please talk about your problems.

3. To what extent did you follow COVID-19 news?

4. How did the news affect your life?
